# Energy Metabolism during Anchorage-Independence. Induction by Osteopontin-c

**DOI:** 10.1371/journal.pone.0105675

**Published:** 2014-08-26

**Authors:** Zhanquan Shi, Bo Wang, Tafadzwa Chihanga, Michael A. Kennedy, Georg F. Weber

**Affiliations:** 1 University of Cincinnati Academic Health Center, Cincinnati, Ohio, United States of America; 2 Department of Chemistry and Biochemistry, Miami University, Oxford, Ohio, United States of America; Institute of Hepatology, Foundation for Liver Research, United Kingdom

## Abstract

The detachment of epithelial cells, but not cancer cells, causes anoikis due to reduced energy production. Invasive tumor cells generate three splice variants of the metastasis gene osteopontin, the shortest of which (osteopontin-c) supports anchorage-independence. Osteopontin-c signaling upregulates three interdependent pathways of the energy metabolism. Glutathione, glutamine and glutamate support the hexose monophosphate shunt and glycolysis and can feed into the tricarboxylic acid cycle, leading to mitochondrial ATP production. Activation of the glycerol phosphate shuttle also supports the mitochondrial respiratory chain. Drawing substrates from glutamine and glycolysis, the elevated creatine may be synthesized from serine via glycine and supports the energy metabolism by increasing the formation of ATP. Metabolic probing with N-acetyl-L-cysteine, L-glutamate, or glycerol identified differential regulation of the pathway components, with mitochondrial activity being redox dependent and the creatine pathway depending on glutamine. The multiple skewed components in the cellular metabolism synergize in a flow toward two mechanisms of ATP generation, via creatine and the respiratory chain. It is consistent with a stimulation of the energy metabolism that supports anti-anoikis. Our findings imply a coalescence in cancer cells between osteopontin-a, which increases the cellular glucose levels, and osteopontin-c, which utilizes this glucose to generate energy.

## Introduction

Anchorage independence is an essential characteristic of metastasizing cells. While untransformed non-hematopoietic cells undergo programmed cell death (anoikis) consecutive to losing contact with their substratum, cancer cells can survive in the circulation for extended periods of time. In fact, the major limiting factor in the process of metastasis formation is the death of the tumor cells before their implantation in the target organs [1]–[3]. Therefore, anchorage independent survival may be more critical to the process of cancer metastasis than organ-specific homing. The molecular mechanisms underlying anchorage independence are poorly understood. The cytokine osteopontin may act as a metastasis gene, particularly through its splice variant osteopontin-c, which has a deletion of exon 4. Osteopontin-c is uniquely expressed in breast cancers, but not in normal breasts [4]. It very effectively supports anchorage independent survival and expansion [5].

Osteopontin-c, but not osteopontin-a, signals through the activation of oxidoreductase gene expression [5] associated with the mitochondrial respiratory chain (NDUFV1, NDUFS4, NDUFS7, NDUFS8, NDUFA9, NDUFB9, Cytochrome c Oxidase), the hexose monophosphate shunt (PGDH) or the regulation of the hexose monophosphate shunt (GPX-4) [5]–[7]. The oxidoreductase induction may lead to the generation of reactive oxygen intermediates in the tumor cells. In fact, it has been reported that escape from anoikis can be mediated through the production of reactive oxygen species, which cause the oxidation and activation of the tyrosine kinase SRC [8], resulting in the transduction of a survival signal.

The detachment of mammary epithelial cells leads to ATP deficiency, owing in part to the loss of glucose transport. Hence, reduced energy production is a feature of anoikis that needs to be overcome in cancer progression [9]. Consistently, increased cancer invasiveness under detached conditions is associated with higher mitochondrial activity, elevated ATP production, pyruvate uptake, and oxygen consumption [10]. We have found that osteopontin-a increases the glucose levels in deadherent breast tumor cells [11], which may provide the biochemical fuel for ATP generation. In conjunction with the observation (referenced above) that osteopontin-c induces oxidoreductases associated with the energy metabolism [5], we investigate the hypothesis that osteopontin-c supports the anchorage independence of cancer cells by upregulating their energy metabolism via redox signaling.

## Materials and Methods

### Reagents, cell lines, DNA constructs and transfection

Poly(2-hydroxyethyl methacrylate) (Poly-HEMA), N-acetyl-L-cysteine (NAC), glutathione (GSH), H_2_O_2_, and mannitol came from Sigma-Aldrich. MCF-7 cells and their transfectants were grown in alpha-MEM with insulin and 10% fetal bovine serum. MCF-7 cells stably transfected with osteopontin-a, osteopontin-c, or vector control have been previously described [5]. ZR-75 cells were grown in RPMI-1640 medium with 10% fetal bovine serum. They were stably transfected with osteopontin-c or vector control in pCR3.1 (selected in G418). The construct for expression of catalase targeted to the mitochondria was obtained from DR. J.A. Melendez [12]. MCF-7 cells were transfected with the FuGENE reagent (Roche) and stable clones were selected in zeocin.

### Immunoblot assay

For the analysis of secreted osteopontin, serum-free cell culture supernatant was collected from each transfectant. 40 µl of supernatant per sample were electrophoresed on 10% SDS-polyacrylamide mini-gels with non-reducing sample buffer. For the analysis of intracellular osteopontin, the cells were lysed in RIPA buffer (50 mM Tris-HCl pH 7.5, 150 mM NaCl, 1% NP-40, 0.5% Na-deoxycholate, 0.1% sodium dodecyl sulfate). Cell lysates at equal amounts of protein (20 µg/lane) were electrophoresed on reducing 10% SDS-polyacrylamide gels. The separated proteins were transferred to PVDF membranes and probed with antibody O-17 (Assay Designs Inc.) to osteopontin. The expression levels of all transfected genes were confirmed every time after cell thawing and initiation of culture.

### Analysis of growth rates

For the investigation of cell growth rates, each cell line was plated at 5000 cells/well in 24-well plates. Daily, five wells per group were harvested by trypsinization and the cell numbers were determined in a Coulter™ Z-Series Counter. At each time point, the cell numbers from the five wells of the various groups of transfectants were analyzed for statistically significant differences by the Wilcoxon-Mann-Whitney test and the t-test, accepting a probability of error of less than 5%.

### Soft agar colony formation

1×10^5^ cells per 60-mm dish were plated in triplicates with a top layer of 0.3% agar (BACTO Agar, Difco, Detroit, MI) and a bottom layer of 0.5% agar (both in alpha-MEM). Every other day, 0.4 ml of medium was supplemented and the plates were examined microscopically for growth. After one week, photographs were taken at high and low magnification and the surface area of all clones in five fields was measured with the imaging software Metamorph (http://www.moleculardevices.com/products/software/meta-imaging-series/metamorph.html).

### Deadhesion in poly-HEMA

To assess the osteopontin splice variant-induced protection from cell death or cell cycle arrest through deadhesion, we plated cells on 0.4 µg/mm^2^ poly-HEMA for 2 days [13], [14]. We then harvested them for gene expression analysis by RNASeq or metabonomic analysis by NMR.

### Metabonomic analysis

We plated MCF-7 transfectants of osteopontin-c, osteopontin-a and vector on poly-HEMA. The cells were harvested (because of deadhesion no trypsin was needed), spun, and the pellet was frozen. Extraction for metabonomics analysis was done as described [17]. NMR spectra were binned buckets with a manual pattern (after removal of regions subject to imperfect water suppression). Total intensity normalization was applied after binning. Initially, unsupervised principal component analysis (PCA) was performed without considering the class information. Statistical significance analysis of the loadings data was performed using Amix (Bruker Biospin, Billerica, MA) based on a published procedure [15], [16], except that a Kruskal-Wallis test was used instead of the Mann-Whitney U test for non-parametric analysis of datasets that were not normally distributed. The bucketing parameters are identical to those used for PCA. This approach has been previously successfully applied to identify changes in lactate, pyruvate, and related metabolites in cancer cells [17].

### ATP assay

6-well dishes were coated with poly-HEMA. MCF-7 transfected cells with vector, osteopontin-a, osteopontin-c, or osteopontin-a plus osteopontin-c were seeded at 4×10^5^ cells per well in complete medium and stayed for two days under standard culture condition. The cells were harvested and homogenized in 100 µl assay buffer, and the ATP levels were measured by a colorimetric assay following the protocol of ATP assay kit (Abcam).

### RNASeq

The Ovation RNA-Seq system (NuGen) was used to initiate amplification at both 3′ end as well as randomly throughout the transcriptome in the sample. 100 ng of total RNA with RIN<5.0, was converted into a library of template molecules suitable for subsequent cluster generation and sequencing by Illumina HiSeq. Total RNA was reverse transcribed and converted to double stranded cDNA with a unique DNA/RNA heteroduplex at one end. Nugen's Ribo-SPIA technology was used for isothermal amplification resulting in the rapid generation of cDNA with a sequence complementary to the original mRNA. The cDNA was then double stranded, fragmented to 200 bp using Covaris S2, and a sequencing library generated using Illumina's TruSeq DNA Sample Prep Kit V2 according to standard protocols. The cDNA library was enriched by a limited number of 10 PCR cycles, validated using an Agilent 2100 Bioanalyzer, and quantitated using the Quant-iT dsDNA HS Kit (Invitrogen). Two individually indexed cDNA libraries were pooled and sequenced on Illumina HiSeq to get a minimum of 90 million reads. Libraries were clustered onto a flow cell using Illumina's TruSeq SR Cluster Kit v2.5 and sequenced 50 cycles using TruSeq SBS Kit -HS on HiSeq. The obtained sequence reads were aligned to the genome by using the standard Illumina sequence analysis pipeline.

### Microarray analysis and RT-PCR confirmation

MCF-7 cells were plated in soft agar, in the presence or absence of hydrogen peroxide. After 7 days of soft agar growth, the clone sizes were measured and RNA was extracted with 2 ml TriReagent LS. Following linear amplification and fluorescence labeling, the samples were subjected to microarray analysis as described [5] (see also http://microarray.uc.edu) using the human 70-mer oligonucleotide library version 2.0 (21,329 optimized oligos) (Qiagen, Alameda, CA) printed on aminosilane-coated slides (Cel Associates, Inc., Pearland, TX). The data representing background-subtracted spot intensities were analyzed after log-transformation and data centering. Statistical significance of differential expression was assessed according to p-values, and adjusting for multiple hypotheses testing by calculating False Discovery Rates (FDR). Estimates of fold-change were calculated, and the cutoff used for significance was a fold change of >2, an intensity of >100, and an FDR<0.05. Significantly changed transcripts were tested against functional assignments with Database for Annotation, Visualization and Integrated Discovery (DAVID). Fisher's Exact Probability, using the Benjamini FDR adjustment, was calculated for each gene category.

The microarray results were validated by real-time PCR [18], which was conducted using a Cepheid Smart Cycler and a SYBR Green detection format. Oligo-dT primed first strand cDNA was synthesized using Invitrogen SuperScript according to the manufacturer's protocol. The PCR reaction contained 0.5× SYBR Green (Roche Diagnostics), MgCl_2_ and primer concentration were optimized for each gene. A 40 cycles PCR protocol consisted of 94°C melting for 15 s, specific annealing for 30 s, and 20 s extension at 72°C. Melt curves yielded a single peak in all cases with no primer dimers. A no-template control was included in all reactions. The levels of β-Actin were used to normalize for equal amounts of RNA.

### Human tumor analysis

The experiments were performed under IRB approval (24-1-29-1, exemption 4) from the Institutional Review Board Medical Center at the University of Cincinnati. Real-time reverse transcription-PCR was done on cDNA from 22 human breast cancer tissues [4]. Primers were designed using Primer3 (http://biotools.umassmed.edu/bioapps/primer3_www.cgi) and constituted NDUFV1 forward 5′-GACTCCCTGTGGGAGATCAG-3′ and reverse 5′-CGAAAGTGGCGGATCAGA-3′ (product size 167 bp), NDUFS7 forward 5′-TGCCCGTGGACATCTACATC-3′ and reverse 5′-CTCCCGCTTGATCTTCCTCT-3′ (product size 152 bp), GPX4 forward 5′-CTTCCCGTGTAACCAGTTCG-3′ and reverse 5′-CGGCGAACTCTTTGATCTCT-3′ (product size 125 bp), PGDH forward 5′-TCTCAATTATGGTGGCATCG-3′ and reverse 5′-GGTTTCGATCAAATGCATCC-3′ (product size 155 bp). The amplification started with 95°C for 1 min, proceeded through 40 cycles of 95°C for 15 sec, 53°C for 30 sec, 68°C for 30 sec, and concluded in 68°C for 5 min, 4°C hold.

Pearson's product moment correlational analysis was applied to assess the correlation between breast tissue osteopontin-c and specific oxidoreductases. Pearson's correlation is a parametric test which assesses the linear association between two continuous variables. Values of the correlation coefficient can range between −1 and 1, with a correlation of 0 representing no correlation between a pair of variables. A coefficient of −1 represents a perfect indirect (negative) correlation. A coefficient of 1 represents a perfect direct (positive) correlation. According to the standard set by Cohen, the guidelines for interpretation of the strength of magnitude for a correlation coefficient are: small  =  absolute value of correlation coefficient between 0.10 and 0.29, medium  =  between 0.30 and 0.49, and large  =  between 0.50 and 1.0.

## Results

### Peroxide signaling supports anchorage independence

Osteopontin exerts strong effects on the redox status of various cells and it prevents programmed cell death in response to diverse stimuli [6], [19], [20]. Based on these observations, we had previously hypothesized that the induction of oxidoreductases by osteopontin-c reflects an anti-oxidant mechanism that protects the cells from anoikis in soft agar [5]. However, in apparent conflict with this notion, there are literature reports indicating that peroxide signaling induces anchorage independence [21]–[24], and that hydrogen peroxide is important in metastasis [25], [26]. We corroborated that reactive oxygen species do support the soft agar colony formation by MCF-7 cells (Supplement S1).

### Osteopontin-c and peroxide signaling converge on common intermediates

**Figure 1:**
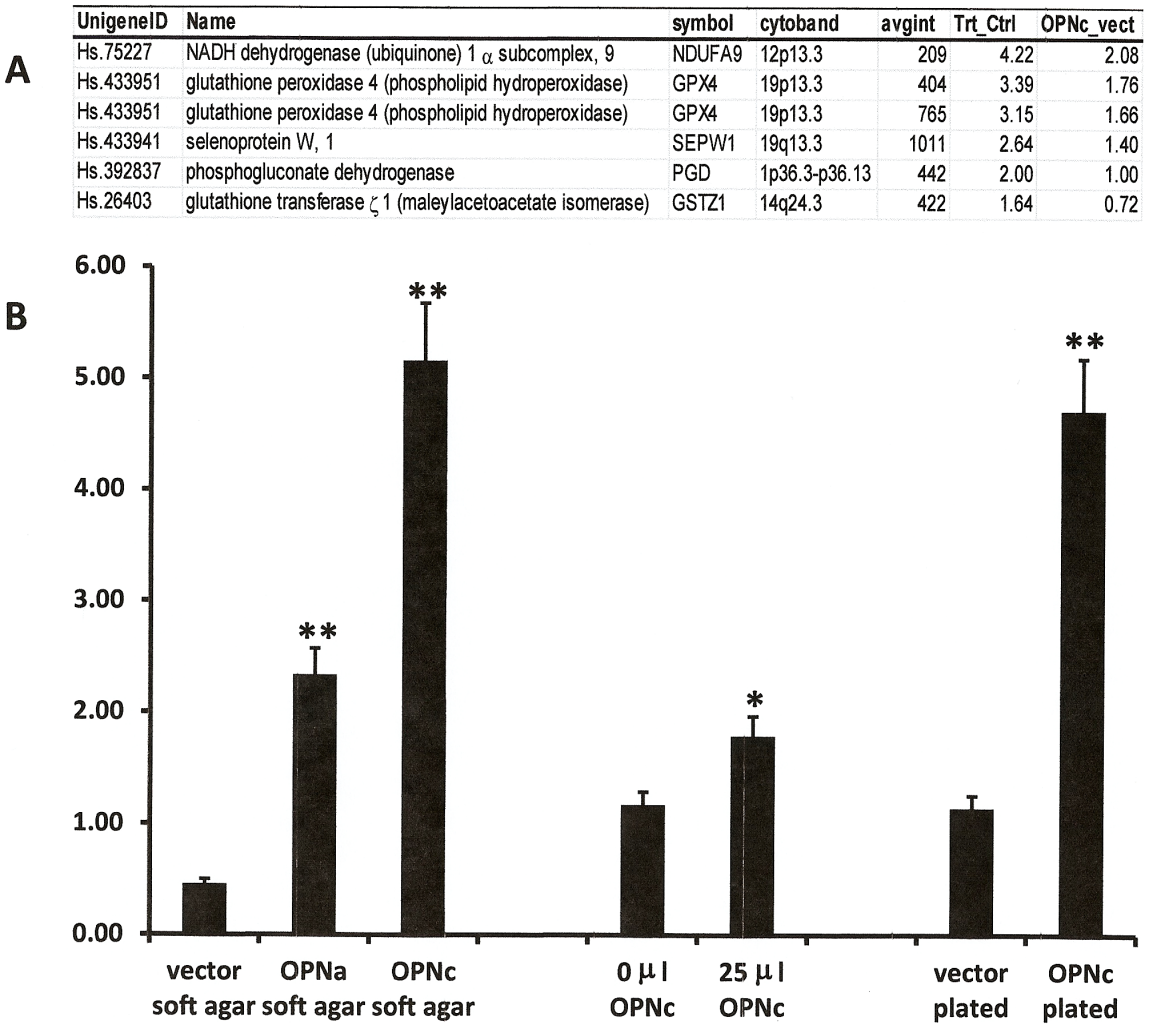
Peroxide signaling is activated by osteopontin-c. A) Oxidoreductase genes commonly induced by osteopontin-c and hydrogen peroxide in soft agar. Microarray analysis after MCF-7 cells had been grown in soft agar for 7 days with or without treatment with hydrogen peroxide. The results are compared to oxidoreductase genes induced by osteopontin-c. T,Trt  =  treated with hydrogen peroxide; C, Ctrl  =  untreated MCF-7 cells; p  =  p-value; avgint  =  average intensity. Note that GPX-4 was represented twice on the array. **B**) Induction of GPX-4 mRNA by osteopontin-c. RNA was extracted from transfected MCF-7 cells after 7 days of soft agar clone formation, from MCF-7 cells plated on plastic dishes and treated with supernatant from MCF-7 OPNc cells, or from transfected MCF-7 cells grown in plastic dishes. GPX-4 mRNA levels were measured compared to actin. MDA-MB-435 cells served as a reference and the relative abundance was calculated [18]. * indicates p<0.05, ** indicates p<0.01 compared to the matching control (in soft agar, the significant difference between MCF-7 OPNa and MCF-7 OPNc is also p<0.01).

Because osteopontin-c and hydrogen peroxide both induce anchorage-independent colony formation, we reasoned that they likely converge on a final common pathway that is also critical for cancer metastasis. To identify common mechanisms between osteopontin-c induced and hydrogen peroxide induced soft agar clone formation, we evaluated the microarray data from soft agar clones of MCF-7 cells either treated with hydrogen peroxide (compared to untreated controls) or transfected with osteopontin-c (compared to vector transfectants) and asked whether any of the oxidoreductases induced by osteopontin-c were also significantly upregulated by hydrogen peroxide. Five genes were identified (Figure 1A). Specifically, we found that GPX-4 is upregulated by both stimuli. We further investigated the GPX-4 RNA levels induced by osteopontin-c using real-time RT-PCR. Transfected osteopontin-c induces the expression of this gene in soft agar as well as in cells grown on plastic dishes. Adding supernatant from MCF-7 OPNc cells (but not supernatant from MCF-7 pCR3.1 cells) was sufficient to cause some induction of GPX-4 expression in MCF-7 cells (Figure 1B). The previously obtained microarray results [5] were confirmed by real-time RT-PCR of four target genes (Supplement S2).

As cells cannot be cleanly extracted from soft agar, we used plating on poly-HEMA as an alternative assay for anchorage-independence. We first confirmed by RNASeq the equivalence of poly-HEMA culture (GEO accession number GSE55193) to soft agar culture [5]. Expectedly, gene ontology analysis revealed the upregulation of numerous redox regulators by osteopontin-c (compared to vector-transfected MCF-7 cells) (Table 1, Supplement S3), but not by osteopontin-a (not shown), confirming that the metabolic effects are comparable to those in soft agar.

**Table 1:** Osteopontin-c induces redox-related gene expression in deadherent cells.

categoryID	description	nGenes	zScore	pValue	FDR	
**GO:0005833**	**hemoglobin complex**	**12**	**38.709**	**0**	**0**	
**GO:0030492**	**hemoglobin binding**	**5**	**38.689**	**0**	**0**	
**GO:0005344**	**oxygen transporter activity**	**13**	**37.064**	**5.44E-301**	**1.44E-297**	
**GO:0015671**	**oxygen transport**	**10**	**29.675**	**8.04E-194**	**1.06E-190**	
GO:0048670	regulation of collateral sprouting	7	27.657	1.16E-168	1.31E-165	
GO:0048668	collateral sprouting	9	24.365	2.03E-131	2.01E-128	
**GO:0015669**	**gas transport**	**15**	**24.215**	**7.79E-130**	**6.86E-127**	
GO:0046852	positive regulation of bone remodeling	10	23.132	1.11E-118	8.00E-116	
**GO:0019825**	**oxygen binding**	**35**	**22.519**	**1.35E-112**	**8.90E-110**	
**GO:0042744**	**hydrogen peroxide catabolic process**	**19**	**21.858**	**3.23E-106**	**1.97E-103**	
GO:0046697	decidualization	17	17.763	6.82E-71	3.38E-68	
GO:0034105	positive regulation of tissue remodeling	17	17.666	3.81E-70	1.78E-67	
**GO:0045429**	**positive regulation of nitric oxide biosynthetic process**	**30**	**16.950**	**9.60E-65**	**4.23E-62**	
**GO:0042743**	**hydrogen peroxide metabolic process**	**35**	**16.763**	**2.29E-63**	**9.55E-61**	
GO:0001893	maternal placenta development	21	15.977	9.28E-58	3.68E-55	
GO:0048640	negative regulation of developmental growth	22	15.520	1.28E-54	4.81E-52	
**GO:0004601**	**peroxidase activity**	**38**	**15.386**	**1.02E-53**	**3.53E-51**	
**GO:0016684**	**oxidoreductase activity, acting on peroxide as acceptor**	**38**	**15.386**	**1.02E-53**	**3.53E-51**	
GO:0046850	regulation of bone remodeling	24	15.049	1.74E-51	5.52E-49	
**GO:0045428**	**regulation of nitric oxide biosynthetic process**	**40**	**14.950**	**7.77E-51**	**2.37E-48**	
**GO:0070301**	**cellular response to hydrogen peroxide**	**42**	**14.813**	**6.00E-50**	**1.76E-47**	
GO:0031103	axon regeneration	28	14.739	1.81E-49	5.13E-47	
GO:0033280	response to vitamin D	26	14.411	2.21E-47	6.05E-45	
GO:0007566	embryo implantation	29	13.634	1.26E-42	3.33E-40	
GO:0031102	neuron projection regeneration	33	13.581	2.61E-42	6.68E-40	
**GO:0006809**	**nitric oxide biosynthetic process**	**49**	**13.435**	**1.88E-41**	**4.52E-39**	
GO:0050840	extracellular matrix binding	33	12.747	1.61E-37	3.75E-35	
GO:0051291	protein heterooligomerization	52	12.678	3.91E-37	8.85E-35	
GO:0034103	regulation of tissue remodeling	34	12.582	1.33E-36	2.93E-34	
**GO:0016209**	**antioxidant activity**	**57**	**12.482**	**4.70E-36**	**1.01E-33**	
GO:0050771	negative regulation of axonogenesis	34	12.407	1.20E-35	2.49E-33	
GO:0045453	bone resorption	38	12.313	3.84E-35	7.81E-33	
**GO:0046209**	**nitric oxide metabolic process**	**60**	**12.090**	**5.98E-34**	**1.19E-31**	
**GO:0034614**	**cellular response to reactive oxygen species**	**63**	**11.996**	**1.87E-33**	**3.61E-31**	
**GO:0020037**	**heme binding**	**128**	**11.950**	**3.24E-33**	**6.12E-31**	
GO:0046906	tetrapyrrole binding	136	11.628	1.49E-31	2.74E-29	
**GO:0042542**	**response to hydrogen peroxide**	**77**	**10.949**	**3.36E-28**	**6.06E-26**	
GO:0048678	response to axon injury	52	10.859	9.07E-28	1.60E-25	
GO:0010811	positive regulation of cell-substrate adhesion	45	10.764	2.54E-27	4.38E-25	
GO:0061387	regulation of extent of cell growth	45	10.694	5.46E-27	9.20E-25	
GO:0090066	regulation of anatomical structure size	245	10.638	9.94E-27	1.64E-24	
GO:0046849	bone remodeling	51	10.540	2.81E-26	4.54E-24	
GO:0032846	positive regulation of homeostatic process	62	10.039	5.16E-24	8.18E-22	
**GO:0034599**	**cellular response to oxidative stress**	**91**	**10.002**	**7.45E-24**	**1.16E-21**	
GO:0008361	regulation of cell size	66	9.960	1.14E-23	1.74E-21	
**GO:0000302**	**response to reactive oxygen species**	**109**	**9.597**	**4.12E-22**	**6.16E-20**	
**GO:0072593**	**reactive oxygen species metabolic process**	**107**	**9.535**	**7.52E-22**	**1.10E-19**	
**GO:0005506**	**iron ion binding**	**217**	**9.466**	**1.46E-21**	**2.10E-19**	

Gene ontology (GO) categories of RNASeq show the gene expression changes in poly-HEMA induced by transfected osteopontin-c. Redox-relevant categories are highlighted in bold. The prevalence in redox-active gene products suggests that the gene expression changes induced by deadhesion on poly-HEMA are equivalent to the gene expression changes induced by deadhesion in soft agar [5]. nGenes  =  number of genes in the group, FDR  =  false discovery rate.

### Osteopontin-c enhances cellular energy production

**Figure 2:**
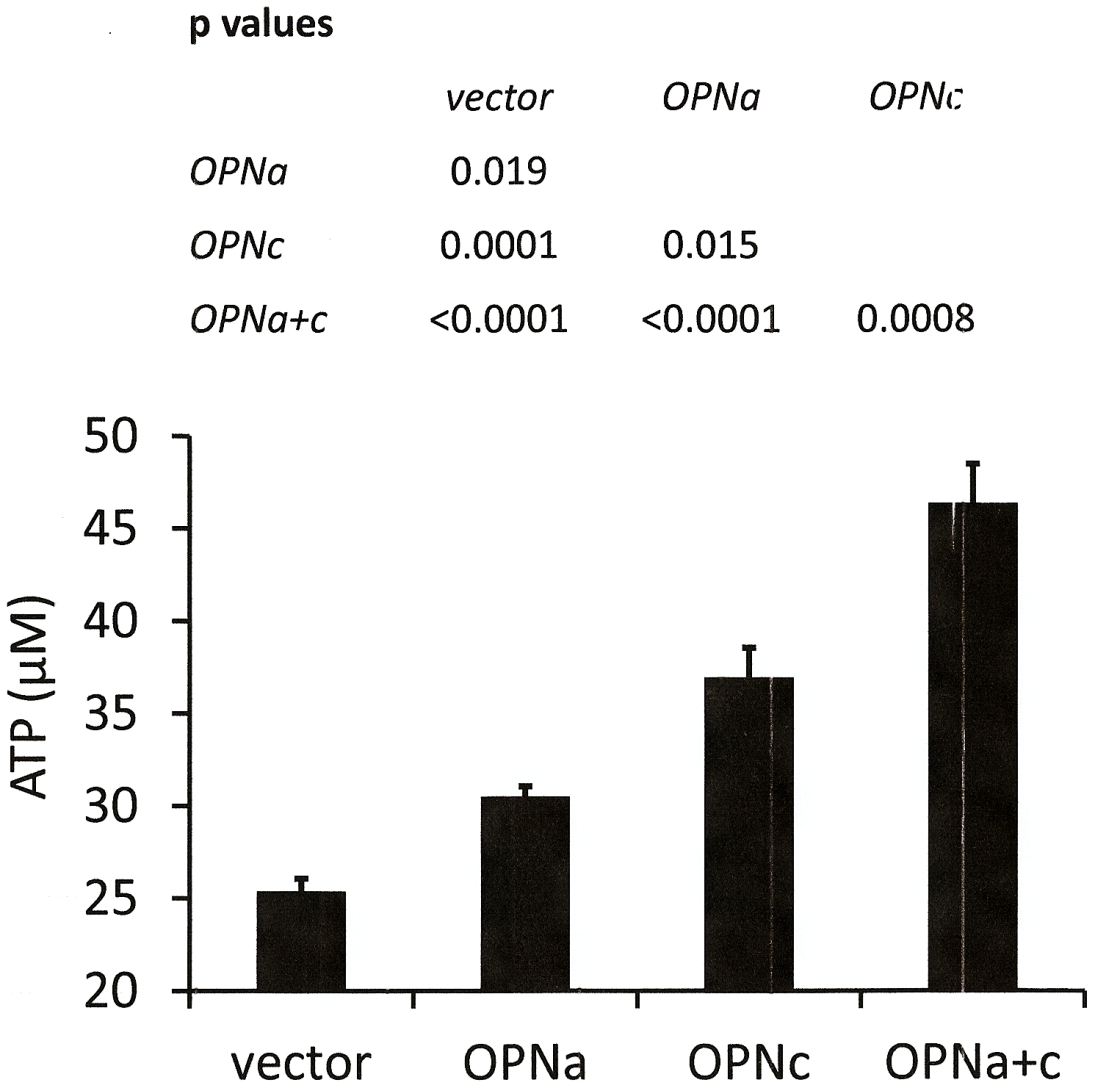
ATP levels in anchorage-independent cells are induced by osteopontin. MCF-7 cells transfected with vector, osteopontin-a, osteopontin-c, or osteopontin-a plus -c were plated on poly-HEMA before colorimetric measurement of the cellular ATP levels. The graph shows mean values (of 3–5 replicates) + sem. The p-values were calculated by one-way analysis of variance (ANOVA). The data represent one experiment of two.

Osteopontin-c is never expressed without osteopontin-a. We have shown that osteopontin-a upregulates the cellular glucose levels, which suggests a model according to which osteopontin-a mobilizes the glucose that is then utilized by osteopontin-c to generate cellular energy in the form of ATP [11]. We therefore measured ATP in deadherent MCF-7 cells, single-transfected with either osteopontin form alone or double-transfected with osteopontin-a plus -c. The ATP levels were significantly higher in osteopontin-c cells than in osteopontin-a cells, and higher in osteopontin-a cells than in vector controls. The double-transfected cells had the highest ATP levels (Figure 2), supporting the hypothesis of a synergy between osteopontin-a and osteopontin-c signaling.

### Osteopontin-c induces redox-sensitive and redox-insensitive metabolic changes

**Figure 3:**
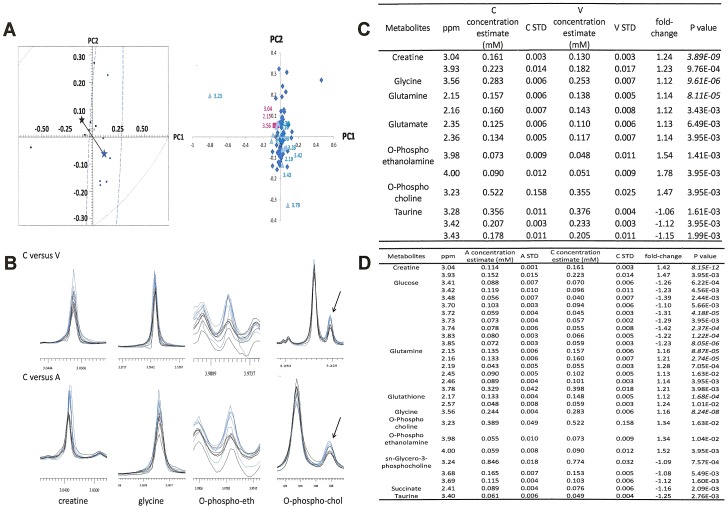
Osteopontin-c supports anchorage-independence through distinct metabolic changes. A) Pair wise comparison of the metabolic profiles induced by osteopontin-c versus vector control. For the osteopontin-c versus osteopontin-a graphs see [11]. The graph on the left shows the score plot of principal component analysis with osteopontin-c samples in black and osteopontin-a samples in blue. The asterisks indicate the centers of the corresponding clusters with the same color. The black line connecting the asterisks represents the group distance (Mahalanobis distance = 1.98). The Mahalanobis distance is calculated using a method previously reported [16]. The graph on the right is the loading plot. The colors indicate significant buckets after statistical analysis. The red squares are filtered by Bonferroni correction and the green triangles are corrected by Benjamini-Hochberg false discovery rate. **B**) Example traces of key metabolites for MCF-7 OPNc cells (blue) compared to MCF-7 vector cells (black). O-phospho-eth  =  O-phosphoethanolamine, O-phospho-chol  =  O-phosphocholine (the arrow points to the metabolite peak). **C**) Summary of metabolites changed by osteopontin-c versus vector control. The table shows ppm values (indicating the center values of the peaks), concentration estimates as the average values of 6 replicates, the standard deviations for six replicates of osteopontin-c (C) or vector (V), and the -fold change for the comparisons between osteopontin-c and vector control. Negative numbers indicate that the metabolite is higher in vector controls than in osteopontin-c transfected cells. Bonferroni correction is in italics, and Benjamini/Hochberg false discovery rate is non-italicized. **D**) Summary of metabolites changed by osteopontin-c versus osteopontin-a (for osteopontin-a versus vector see [11]). The -fold change is calculated for comparisons between osteopontin-c (C) and osteopontin-a (A). Negative numbers indicate that the metabolite is higher in osteopontin-a transfected cells than in osteopontin-c transfected cells. Bonferroni correction is in italics, and Benjamini/Hochberg false discovery rate is non-italicized.

Many of the oxidoreductases induced by osteopontin-c [5] belong to the mitochondrial respiratory chain or to the hexose monophosphate shunt or regulate the hexose monophosphate shunt. Therefore, biochemical processes associated with the intermediary metabolism for energy generation are likely to be important mediators of the osteopontin-c effects on breast tumor cells that have lost contact with the substratum (for supportive evidence see Supplement S4). The metabolite profile of deadherent MCF-7 transfectants, derived from NMR analysis, identified glutathione, glutamine, glutamate, creatine, glycine, O-phosphocholine, and O-phosphoethanolamine to be induced by osteopontin-c (Figure 3). Aspartate and alanine are moderately increased (not shown). Taurine is suppressed by osteopontin-c and osteopontin-a, which is reflected in an apparent upregulation in vector compared to both forms of osteopontin. Taurine can act as an antioxidant [27] and as a mitochondrial matrix pH buffer [28]. Its downregulation may reflect taurine consumption by the increased mitochondrial redox activity. Similar results were obtained with ZR-75 cells (Table 2).

**Table 2:** Metabolic effects of osteopontin-c in ZR-75 cells.

ZR-75 cells				C vs V	
Metabolites	ppm	C STD	V STD	fold-change	p-value
alanine	1.485	3.63E-03	1.98E-03	1.50	9.02E-03
glutamate	2.065	6.30E-04	4.69E-04	1.08	9.02E-03
glutamate	2.34	7.53E-04	1.14E-03	1.56	1.58E-06
glutamate	2.35	1.18E-03	9.56E-04	1.57	1.68E-07
glutamate	2.36	9.45E-04	4.61E-04	1.56	2.94E-08
glutamate	2.37	5.88E-04	9.56E-04	1.43	3.37E-06
aspartate	2.642	7.19E-04	5.76E-04	1.35	9.02E-03
aspartate	2.801	1.04E-03	6.20E-04	1.06	3.17E-01
aspartate	2.829	6.98E-04	5.35E-04	1.02	5.10E-01
creatine	3.04	1.62E-03	5.02E-04	1.65	3.67E-08
taurine	3.424	7.61E-04	8.55E-04	−1.48	1.96E-09
glycine	3.562	4.82E-03	7.07E-04	1.96	6.46E-07
creatine	3.932	1.19E-03	4.48E-04	1.21	2.22E-06
o-phosphoethanolamine	4.008	2.07E-03	8.46E-04	1.87	9.02E-03
o-phosphoethanolamine	4.068	1.11E-03	4.00E-04	2.99	1.22E-08

ZR-75 breast tumor cells, stably transfected with osteopontin-c (C) or vector control (V), were cultured in poly-HEMA coated plates for 48 hours and metabonomics analysis was performed on their lysates.

To assess the specificity of the metabolic osteopontin-c effects, we tested the contributions by the amino acid sequence around the OPN-c splice junction. By site-directed mutagenesis, we generated the mutant OPNcM3 with the internal sequence SGSSAAAAAAASSEET (altered from the splice junction SGSSEEKQNAVSSEET). In OPNcM3, all the amino acids around the splice junction between the proximal N-terminal and C-terminal serines are alanines. If the splice junction was critical to the osteopontin-c dependent metabonomic changes, the metabolite profile of deadherent MCF-7 OPNcM3 cells was expected to resemble MCF-7 vector cells. Indeed, the fold changes of osteopontin-c transfectants to osteopontin-c M3 transfectants (Table 3) are equal to the comparison of osteopontin-c to vector (see Figure 3), confirming a loss of function by the site-directed mutagenesis. Unexpectedly, one metabolite (glutamate) was not susceptible to the osteopontin-c mutation (not shown). The reason for this resistance is unknown.

**Table 3:** The metabolic effects of osteopontin-c depend on the splice junction.

				C vs M3	
Metabolites	ppm	C STD	M3 STD	fold-change	p-value
creatine	3.036	2.76E-03	4.18E-03	1.30	4.10E-06
glycine	3.561	3.08E-03	4.46E-03	1.05	3.56E-02
creatine	3.932	2.85E-03	2.88E-03	1.40	4.91E-08
o-phosphoethanolamine	3.993	1.48E-03	4.10E-03	1.27	8.58E-05
glutamine	2.449	8.10E-04	1.49E-03	1.07	4.21E-03
aspartate	2.814	1.06E-03	1.24E-03	1.15	2.84E-03
aspartate	2.674	6.45E-04	3.04E-03	1.26	2.53E-02
glutamate	2.355	8.13E-04	1.09E-03	-1.15	1.83E-08
taurine	3.421	2.56E-03	3.19E-03	-1.29	1.60E-05

The metabolic effects of osteopontin-c depend on the splice junction. We stably transfected MCF-7 cells with OPNc versus OPNcM3. Expression was confirmed by Western blotting [44]. MCF-7 cells transfected with osteopontin-c or its mutant M3 were plated in poly-HEMA coated wells. After 48 hours, the cells were harvested and lysed for analysis of the metabolite levels by NMR. Shown are the fold-change and p-value for the steady state-levels of metabolites in osteopontin-c transfectants (C) versus mutant transfectants (M3).

To further assess the relevance of redox processes in the osteopontin-c dependent metabolic changes, we repeated the NMR measurements for MCF-7 OPNc versus MCF-7 vector cells on poly-HEMA and included a sample of MCF-7 OPNc cells treated with 4 mM N-acetyl L-cysteine (NAC). While NAC did not affect the upregulation of creatine, O-phosphoethanolamine, and O-phosphocholine, it partially reduced the osteopontin-c dependent upregulation of glutamine and downregulation of taurine. Remarkably, the osteopontin-c mediated increases in glutamate, aspartate, and alanine, were suppressed by NAC below the baseline constituted by MCF-7 vector (Table 4). This is consistent with the need for some redox activity in all cases of anchorage-independent survival (compare Supplement S1). However, the results also corroborate the unique association of certain redox-sensitive metabolic intermediates, specifically linked to mitochondrial activity, with the osteopontin-c dependent pathways.

**Table 4:** Effect of NAC on the metabolic changes induced by osteopontin-c on deadherent cells.

							C control vs V		C NAC vs V		C control vs C NAC	
ppm	C concentration (mM)	C STD	C NAC concentration (mM)	C NAC STD	V concentration (mM)	V STD	fold-change	p-value	fold-change	p-value	fold-change	p-value
1.478	0.099	1.11E-03	0.063	4.81E-04	0.089	1.47E-03	1.11	7.71E-02	−1.42	3.39E-02	1.58	4.95E-02
1.49	0.105	6.67E-04	0.065	4.21E-04	0.091	1.85E-03	1.15	1.57E-01	−1.4	3.39E-02	1.61	4.95E-02
2.694	0.016	9.14E-05	0.011	7.64E-05	0.013	1.46E-04	1.23	3.39E-02	−1.24	3.39E-02	1.53	4.95E-02
2.707	0.016	2.56E-04	0.012	8.72E-05	0.014	6.62E-05	1.15	3.39E-02	−1.16	3.39E-02	1.34	4.95E-02
2.797	0.016	2.37E-04	0.011	5.27E-05	0.014	9.21E-05	1.17	3.39E-02	−1.22	3.39E-02	1.43	4.95E-02
2.803	0.017	8.93E-05	0.011	1.31E-04	0.013	1.31E-04	1.25	3.39E-02	−1.24	3.39E-02	1.55	4.95E-02
2.826	0.015	7.40E-05	0.011	4.79E-05	0.013	5.74E-05	1.16	3.39E-02	−1.16	3.39E-02	1.34	4.95E-02
2.832	0.015	1.32E-04	0.011	6.11E-05	0.013	1.15E-04	1.14	7.71E-02	−1.17	3.39E-02	1.33	4.95E-02
3.04	0.123	4.16E-04	0.112	8.83E-05	0.114	3.09E-04	1.08	3.39E-02	−1.02	7.71E-02	1.1	4.95E-02
3.932	0.074	9.36E-04	0.075	4.29E-04	0.071	7.41E-04	1.03	4.80E-01	1.05	1.57E-01	−1.02	5.13E-01
2.337	0.025	2.47E-04	0.019	2.15E-04	0.023	2.84E-04	1.11	7.71E-02	−1.21	3.39E-02	1.34	4.95E-02
2.343	0.028	4.77E-04	0.02	6.28E-05	0.025	3.05E-04	1.15	7.71E-02	−1.24	3.39E-02	1.43	4.95E-02
2.349	0.049	7.98E-04	0.034	3.57E-04	0.039	3.52E-04	1.24	3.39E-02	−1.16	3.39E-02	1.43	4.95E-02
2.356	0.043	6.32E-04	0.031	3.45E-04	0.036	3.30E-04	1.2	3.39E-02	−1.17	3.39E-02	1.4	4.95E-02
2.362	0.029	8.35E-04	0.021	1.38E-04	0.024	2.41E-04	1.22	3.39E-02	−1.17	3.39E-02	1.42	4.95E-02
2.369	0.033	1.05E-03	0.022	2.95E-04	0.027	4.36E-04	1.21	1.57E-01	−1.2	3.39E-02	1.45	4.95E-02
2.45	0.054	1.13E-03	0.048	3.12E-04	0.045	4.53E-04	1.2	3.39E-02	1.06	1.57E-01	1.14	4.95E-02
2.464	0.051	1.03E-03	0.046	5.62E-04	0.043	4.17E-04	1.19	3.39E-02	1.06	1.57E-01	1.13	2.75E-01
2.477	0.025	3.89E-04	0.022	3.09E-04	0.02	3.37E-04	1.25	3.39E-02	1.1	1.57E-01	1.13	2.75E-01
3.562	0.147	3.88E-03	0.118	2.05E-04	0.137	1.11E-03	1.07	4.80E-01	−1.16	3.39E-02	1.24	4.95E-02
3.225	0.146	4.59E-03	0.173	3.77E-03	0.103	1.43E-03	1.41	3.39E-02	1.68	3.39E-02	−1.19	2.75E-01
3.973	0.018	1.75E-04	0.02	3.57E-04	0.016	2.88E-04	1.16	7.71E-02	1.23	1.57E-01	−1.07	5.13E-01
3.98	0.047	6.27E-04	0.046	4.88E-04	0.035	1.40E-03	1.34	3.39E-02	1.31	3.39E-02	1.02	5.13E-01
3.999	0.02	1.32E-04	0.019	1.48E-04	0.016	4.35E-04	1.31	3.39E-02	1.22	3.39E-02	1.07	1.27E-01
3.258	0.02	5.55E-04	0.023	2.51E-04	0.025	4.63E-04	−1.28	1.57E-01	−1.1	1.57E-01	−1.17	2.75E-01
3.269	0.042	8.38E-04	0.047	7.48E-04	0.054	1.08E-03	−1.28	7.71E-02	−1.13	1.57E-01	−1.13	2.75E-01
3.411	0.025	4.66E-04	0.028	3.32E-04	0.032	5.26E-04	−1.24	3.39E-02	−1.14	1.57E-01	−1.09	2.75E-01
3.433	0.022	5.93E-04	0.023	2.66E-04	0.025	2.02E-04	−1.15	1.57E-01	−1.08	1.57E-01	−1.06	5.13E-01

MCF-7 cells transfected with osteopontin-c or vector control were plated in poly-HEMA coated wells. 4 mM N-acetyl L-cysteine was added to one sample of MCF-7 OPNc cells. After 48 hours, the cells were harvested and lysed for analysis of the metabolite levels by NMR. Shown are comparison of MCF-7 OPNc (C) to MCF-7 vector (V), comparison of MCF-7 OPNc + NAC to MCF-7 vector, comparison of MCF-7 OPNc to MCF-7 OPNc + NAC.

### Glutamine is a mediator of the osteopontin-c effect

Osteopontin-c increases the level of glutamine in deadherent cells (compare Figure 3). Standard cell culture medium contains 2 mM L-glutamine. To probe for the glutamine-dependent metabolic pathways associated with the osteopontin-c effect we altered the levels of L-glutamine in the medium, under the rationale that its reduction will partially suppress the metabolism in deadherent MCF-7 OPNc cells while its elevation will mimic some of the osteopontin-c effects in deadherent MCF-7 vector cells. The comparison of the metabolic profiles of MCF-7 OPNc cells in 2 mM L-glutamine (standard) to MCF-7 OPNc cells in 0.5 mM L-glutamine (low), to MCF-7 vector cells in 2 mM L-glutamine (standard), to MCF-7 vector cells in 4 mM L-glutamine (high) indicated that L-glutamine is essential for the modulation of taurine levels, and is necessary but not sufficient for the creatine/O-phosphoethanolamine/O-phosphocholine pathway. As glutamine can be converted to glutamate (catalyzed by glutamine synthase, GS) and aspartate (transamination reaction catalyzed by aspartate aminotransferase, AST), its levels correlate with the levels of those products (Table 5).

**Table 5:** Effect of L-glutamine on the metabolism of deadherent cancer cells.

										C 2 mM vs V 2 mM		C 2 mM vs C 0.5 mM		V 4 mM vs V 2 mM	
Metabolites	ppm	C 2 GLU concentration (mM)	C 2 GLU STD	C 0.5 GLU concentration (mM)	C 0.5 GLU STD	V 4 GLU concentration (mM)	V 4 GLU STD	V 2 GLU concentration (mM)	V 2 GLU STD	fold-change	p-value	fold-change	p-value	fold-change	p-value
Alanine	1.478	0.11	3.44E-04	0.109	9.94E-04	0.062	1.06E-03	0.076	2.27E-03	1.45	6.73E-05	1.01	7.49E-01	−1.23	4.33E-02
Alanine	1.49	0.113	4.24E-04	0.112	9.60E-04	0.064	1.07E-03	0.077	2.06E-03	1.46	2.33E-05	1.01	7.99E-01	−1.21	4.33E-02
Aspartate	2.694	0.02	8.44E-05	0.017	7.48E-05	0.02	8.88E-05	0.019	1.58E-04	1.04	7.27E-02	1.15	2.44E-04	1.06	2.11E-02
Aspartate	2.707	0.02	9.34E-05	0.018	8.35E-05	0.021	1.14E-04	0.019	1.85E-04	1.05	1.97E-02	1.16	2.77E-04	1.07	8.11E-03
Aspartate	2.797	0.02	9.66E-05	0.017	1.33E-04	0.02	1.14E-04	0.019	1.30E-04	1.03	1.65E-01	1.15	1.73E-03	1.04	8.87E-02
Aspartate	2.803	0.019	5.10E-05	0.017	1.07E-04	0.02	4.11E-05	0.018	2.23E-04	1.05	2.02E-02	1.12	9.10E-04	1.1	1.23E-03
Aspartate	2.826	0.016	5.23E-05	0.015	9.56E-05	0.016	1.75E-04	0.016	1.37E-04	1.02	3.57E-01	1.09	6.16E-03	1.02	6.60E-01
Aspartate	2.832	0.016	4.43E-05	0.015	3.98E-05	0.017	9.58E-05	0.015	1.70E-04	1.05	4.10E-02	1.07	8.98E-04	1.07	4.33E-02
Creatine	3.04	0.138	3.50E-04	0.144	3.42E-04	0.117	6.94E-04	0.121	5.36E-04	1.14	2.77E-05	−1.04	6.93E-03	−1.03	1.50E-01
Creatine	3.932	0.093	4.93E-04	0.098	3.02E-04	0.083	3.18E-04	0.085	1.89E-04	1.1	5.12E-03	−1.04	4.33E-02	−1.03	2.09E-01
Glutamate	2.337	0.027	1.45E-04	0.024	1.83E-04	0.024	1.83E-04	0.023	2.62E-04	1.19	4.33E-02	1.13	2.09E-02	1.05	4.06E-01
Glutamate	2.343	0.03	1.31E-04	0.026	1.20E-04	0.028	2.01E-04	0.026	1.65E-04	1.16	8.65E-03	1.13	6.32E-04	1.07	1.80E-01
Glutamate	2.349	0.05	1.87E-04	0.045	2.64E-04	0.045	3.51E-04	0.042	2.51E-04	1.2	2.95E-03	1.11	2.09E-02	1.07	1.59E-01
Glutamate	2.356	0.046	2.61E-04	0.04	2.77E-04	0.041	3.61E-04	0.038	1.01E-04	1.22	2.09E-02	1.13	3.25E-03	1.1	4.33E-02
Glutamate	2.362	0.029	1.80E-04	0.026	1.28E-04	0.026	2.57E-04	0.024	1.24E-04	1.2	7.27E-04	1.12	3.49E-03	1.08	9.13E-02
Glutamate	2.369	0.032	2.30E-04	0.029	1.28E-04	0.029	2.88E-04	0.027	5.63E-05	1.18	9.26E-04	1.1	8.06E-03	1.08	7.37E-02
Glutamine	2.45	0.077	4.08E-04	0.03	2.08E-04	0.085	5.35E-04	0.058	1.96E-03	1.33	4.92E-05	2.6	3.82E-08	1.47	1.33E-05
Glutamine	2.464	0.073	3.70E-04	0.028	3.25E-04	0.082	5.54E-04	0.055	1.86E-03	1.31	2.09E-02	2.62	2.09E-02	1.47	2.09E-02
Glutamine	2.477	0.032	1.55E-04	0.014	1.41E-04	0.037	1.98E-04	0.026	7.43E-04	1.24	1.17E-04	2.38	9.45E-08	1.41	1.00E-05
Glycine	3.562	0.063	4.12E-04	0.071	6.67E-04	0.041	2.64E-04	0.048	5.77E-04	1.33	1.65E-05	-1.13	1.42E-02	−1.17	1.40E-04
O-Phosphocholine	3.225	0.136	8.69E-04	0.153	8.32E-04	0.12	7.48E-04	0.122	2.44E-03	1.11	5.91E-02	−1.08	1.27E-04	0.98	4.07E-02
O-Phosphoethanolamine	3.973	0.017	9.78E-05	0.019	2.93E-05	0.014	8.36E-05	0.014	1.22E-04	1.19	2.09E-02	−1.12	4.29E-04	−1.01	2.48E-01
O-Phosphoethanolamine	3.98	0.038	2.36E-04	0.042	1.62E-04	0.025	4.11E-04	0.024	1.06E-04	1.55	3.51E-06	−1.12	1.86E-03	1.02	7.73E-01
O-Phosphoethanolamine	3.999	0.016	1.29E-04	0.018	1.02E-04	0.011	1.13E-04	0.01	1.12E-04	1.63	1.11E-05	−1.09	2.88E-02	1.08	1.29E-01
Taurine	3.258	0.025	1.07E-04	0.029	8.02E-05	0.028	1.16E-04	0.03	1.58E-04	−1.21	2.36E-04	−1.15	7.22E-05	−1.09	1.20E-02
Taurine	3.269	0.065	3.31E-04	0.084	1.36E-04	0.062	3.64E-04	0.072	1.22E-03	−1.1	1.22E-02	−1.29	2.05E-06	−1.15	2.09E-02
Taurine	3.411	0.032	1.71E-04	0.037	1.26E-04	0.034	2.29E-04	0.039	7.33E-04	−1.2	1.05E-03	−1.14	3.18E-04	−1.16	4.19E-03
Taurine	3.433	0.027	1.49E-04	0.031	1.89E-04	0.028	2.25E-04	0.031	2.54E-04	−1.14	8.02E-04	−1.15	1.54E-03	−1.09	1.57E-02

MCF-7 cells transfected with osteopontin-c (C) were plated in poly-HEMA coated wells in complete medium (2 mM L-glutamine) or low glutamine medium (0.5 mM). MCF-7 vector cells (V) were plated in poly-HEMA coated wells in complete medium (2 mM L-glutamine) or high glutamine medium (4 mM). After 48 hours, the cells were harvested and lysed for analysis of the metabolite levels by NMR. Shown are comparison of MCF-7 OPNc to MCF-7 vector, both at normal levels of L-glutamine (2 mM), comparison of MCF-7 OPNc normal to low L-glutamine, comparison of MCF-7 vector normal to high L-glutamine.

### Glycerol transport contributes to the osteopontin-c effect

**Figure 4:**
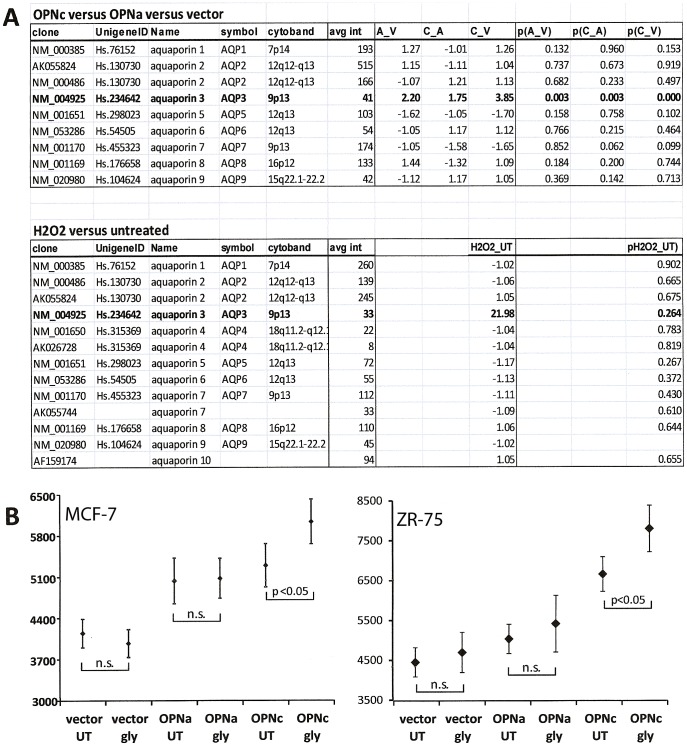
Glycerol modulation of the osteopontin-c effect in cancer cells. A) Inductions of aquaporins by osteopontin or hydrogen peroxide. Microarray analysis was performed after extracting the RNA of either MCF-7 vector, MCF-7 OPNa, and MCF-7 OPNc cells or untreated and hydrogen peroxide-treated MCF-7 cells from soft agar on day 7 after plating. The results were examined for changes in the expression of aquaporins. **B**) Soft agar clone formation by MCF-7 transfectants (left panel) or ZR-75 transfectants [44] (right panel) in the presence or absence of glycerol. The soft agar assays were performed with the addition of 0.1% (v/v) autoclaved glycerol (20 µl at plating for MCF-7 cells, 5 µl at plating for ZR-75 cells) with a maintenance dose of 0.2 µl in 400 µl medium every other day. Glycerol induced the clone size by MCF-7 OPNc cells, but not MCF-7 vector cells in 3 additional experiments (the numbers indicate relative clone sizes). The glycerol-induced increase in clone size for OPNc cells is significant (over untreated OPNc cells) at p<0.05. Each symbol represents the average of 12–23 data points for MCF-7 cells and 11–25 data points for ZR-75 cells. gly  =  glycerol.

Glycerol is important in the regulation of the cellular energy metabolism. It may be transported into cells through aquaporin-3. Although expressed at low levels, this aquaporin is the only one that is significantly upregulated by osteopontin-c and is induced by H_2_O_2_ treatment in soft agar (Figure 4A). We therefore tested the effects of glycerol on soft agar clone formation. The addition of autoclaved glycerol to soft agar assays at plating caused an increase in clone size on day 7 in MCF-7 OPNc and MCF-7 OPNa+OPNc cells (not shown), but not in MCF-7 vector or MCF-7 OPNa cells. Similar results were obtained with transfected ZR-75 cells (Figure 4B), suggesting that glycerol can enhance osteopontin-c signaling.

### Osteopontin-c abundance correlates with oxidoreductase expression in patient specimens

**Figure 5:**
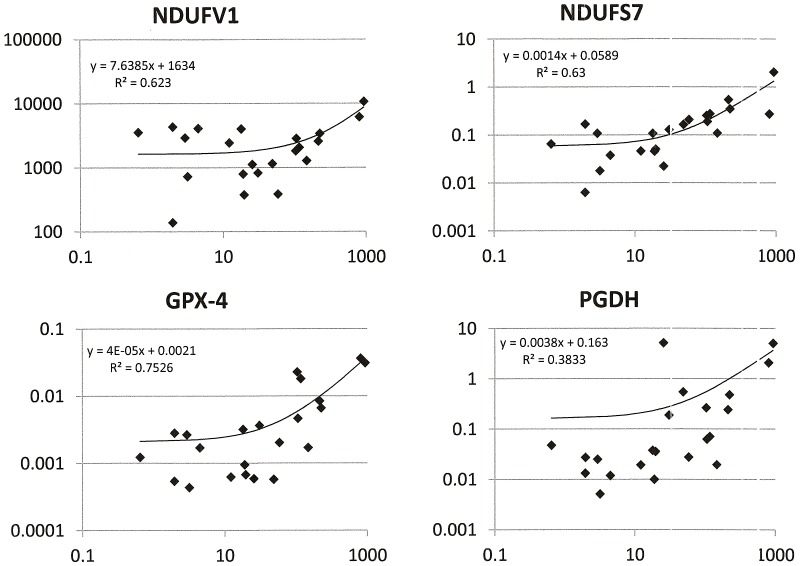
RNA levels of osteopontin-c and oxidoreductase genes in breast cancer tissue. cDNA from 22 human breast cancer specimens [4] was analyzed by real-time RT-PCR for the expression levels of osteopontin-c (x-axes in all graphs) compared to NDUFV1 (top left), NDUFS7 (top right), GPX-4 (bottom left), and PGDH (bottom right). The trend lines in the double-logarithmic plots show the linear correlations between the two gene products, R^2^  =  correlation coefficient. The relative abundance was calculated in relation to the cDNA from a reference cell line (MCF-7 OPNc in soft agar) [18].

The above results show that osteopontin-c induces oxidoreductases in breast cancer cells. To validate our results from cell line experiments we asked whether the expression levels of osteopontin-c RNA correlate with specific oxidoreductases (previously found to be upregulated in the microarray analysis from soft agar) in breast cancer tissue of patients. There was indeed a strong correlation for osteopontin-c with NDUFV1, NDUFS7 and GPX-4, and a moderate correlation with PGDH (Figure 5).

## Discussion

**Figure 6:**
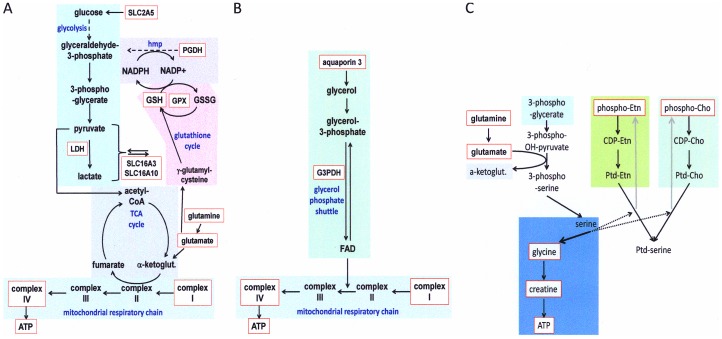
Metabolic pathways affected by osteopontin-c. A) The schema depicts the connections among the biochemical pathways for glutathione cycle, hexose monophosphate shunt (hmp), glycolysis, tricarboxylic acid cycle (TCA cycle), and the respiratory chain. Enzymes (PGDH, GPX4, LDH), transporters (SLC gene products), metabolites (glutamine, glutamate) and complexes (I and IV of the respiratory chain) upregulated by osteopontin-c are consistent with a metabolic shift toward increased mitochondrial respiratory chain activity. The genes and complexes upregulated by osteopontin-c are highlighted in red boxes. Evidence comes from this study, [5], and searches in Oncomine. ketoglut  =  ketoglutarate. **B**) The pathway shows the mode of glycerol utilization via the glycerol phosphate shuttle, which is supported by the biological effects of exogenous glycerol, and the upregulation of an enzyme (G3PDH) and a transporter (aquaporin). Like pathway A), the glycerol phosphate shuttle activates the mitochondrial respiratory chain. **C**) The upregulation of creatine is required for ATP regulation. Creatine is produced from serine via glycine. The depletion of available serine in this process blocks the ethanolamine (Etn) and choline (Cho) pathways (dashed arrows), thus leading to a build-up of phosphoethanolamine and phosphocholine (grey arrows). Metabolites upregulated according to NMR analysis are highlighted in red boxes. Ptd  =  phosphatidyl.

We previously described the support of anchorage-independence in cancer cells by osteopontin-c. We found the phenomenon to be associated with the upregulation of oxidoreductase genes [5]. Here, we have gained mechanistic insight into the osteopontin-c effect. ATP deprivation is associated with deadhesion and leads to anoikis [9]. Cancer cells that have been shed from a primary tumor need to overcome this energy deficit to survive and form metastases. Osteopontin-c signaling protects the deadherent cells from cell death by increasing the generation of ATP. This is reflected in elevated levels of gene products and metabolites associated with the hexose monophosphate shunt, glycolysis, and mitochondrial complexes, which jointly feed into activation of the respiratory chain. The glutathione cycle plays a regulatory role among those pathways, reflected in the upregulation of the substrate glutathione and the induction of the enzyme GPX-4 (Figure 6A). Glycerol directly activates the mitochondrial respiratory chain through the induction of the glycerol-phosphate shuttle (Figure 6B). In a redox-insensitive pathway component that requires glutamine, creatine is synthesized from serine via glycine and supports the energy metabolism by increasing the formation of ATP. The glycolysis intermediate 3-phospho-glycerate likely provides the substrate for serine synthesis. Increased serine pathway flux and glycine utilization have been identified in breast cancer [29], [30]. Our results suggest that much of the synthesized serine may be utilized in creatine production. The depletion of available serine in the ATP-generating pathway may block the ethanolamine and choline pathways, thus leading to the observed build-up of phosphoethanolamine and phosphocholine (Figure 6C). The pathway components outlined in Figures 4A and 4B both converge on the mitochondrial respiratory chain. The pathway components in Figures 4A and 4C are connected via glycolysis and glutaminolysis. Glutamine and glutamate can feed into the glutathione cycle or the tricarboxylic acid cycle. Glutaminolysis could potentially generate 12 molecules of ATP per molecule of glutamine, or its abundance may affect the levels of glutamate and aspartate. Of note, although the present investigation focuses on the selective effects of osteopontin-c, this splice variant is never expressed without the full-length variant osteopontin-a (untransformed breast epithelial cells may express no osteopontin or osteopontin-a alone, 75–80% of breast cancers express osteopontin-a plus osteopontin-c). Our studies of the osteopontin-a effect have suggested that this form elevates the cellular glucose levels, which likely support and enhance the osteopontin-c effect [11]. The results suggest that anchorage-independent survival is achieved in cancer cells when metastasis genes synergize to maintain the metabolic profile associated with the rapidly growing primary tumor cells.

The recent resurgence of interest in cancer cell metabolism, driven in part by innovative technical capabilities, such as mass spectrometry and nuclear magnetic resonance, has provided some insights into hitherto enigmatic aspects of carcinogenesis. Much emphasis has been placed on understanding the Warburg effect, and various explanations have been put forward for its molecular causes [31], [32]. By contrast, teleological explanations for why and how the metabolic changes in cancer cells may benefit their proliferation and dissemination have been limited and largely dissatisfactory. Two visible theories suggest that mitochondrial hyperpolarization leads to anti-apoptosis because low H_2_O_2_ and high NF-AT suppress Kv channels [33] or that anabolic requirements are met if glutamine metabolism generates NADPH, which restores oxaloacetate for the TCA cycle and supports protein and nucleotide synthesis [34]. Both explanations imply metabolic contributions to early stages of transformation. Osteopontin-c acts at later stages, promoting tumor progression. It supports anchorage-independence (a prerequisite for dissemination) and induces metabolic changes that lead to energy generation, manifested in elevated ATP levels. Our results show that an upregulation of the energy metabolism is important in tumor progression and provides a metabolic explanation for the anchorage independent expansion of transformed, but not untransformed cells (the latter do not produce osteopontin-c).

The role of reactive oxygen species in tumorigenesis has not been completely clear. In fact, they may negatively or positively impact transformation. Reactive metabolites of oxygen can have a protective effect on the host through their ability to kill transformed cells. In fact, this is the mechanism of action of various anti-cancer drugs. The cellular abundance of superoxides and lipid peroxides is increased during apoptosis, regardless of the type of triggering stimulus [35], [36], and elevated levels of hydroxyl radical (OH) typically induce programmed cell death [37]. Furthermore, oxidative damage increases in all tissues with aging and may lead to cellular senescence. The senescence-associated loss of functional capacity is due to molecular defects induced by accumulated reactive oxygen intermediates, which escape elimination from the cell by protective enzyme systems, such as catalase [38], [39]. The rate of telomere shortening may be affected (in addition to the history of population doublings) by single strand breaks derived from oxidative stress [40]. Hyperoxia shortens the replicative life span, while low oxygen tension gives rise to an extension of the replicative life span [41], [42]. On the other hand, reactive oxygen species have been considered to pose a risk for cancer due to their oxidative modifications of DNA, which may activate proto-oncogenes or inactivate tumor suppressor genes, in either case resulting in excessive cell cycle progression. Also, oxidative DNA damage leading to a loss of function of DNA repair genes gives rise to genomic and genetic instability, thus facilitating the alteration of genes associated with transformation. Finally, the dissemination of tumor cells is an integral characteristic of advanced stages of transformation. Elevated levels of hydrogen peroxide mediate metabolic changes that allow adhesion-independent survival and consecutive metastatic spread [25], [26]. The metastasis gene osteopontin-c acts as an autocrine inducer.

The distinct impacts by osteopontin-c and the full-length form osteopontin-a on anchorage independence could reflect quantitative differences in signaling induced by the two forms of osteopontin due to different affinities to the same receptors. Alternatively, it could reflect a loss of function by osteopontin-c due to the absence of exon 4, a gain of function of osteopontin-c due to the generation of a unique domain at the splice junction, or both. Exon 4, which is lost in osteopontin-c, contains the transglutamination site and may be responsible for the calcium-dependent aggregation of osteopontin-a, but not osteopontin-c. This implies that osteopontin-c remains soluble, even under conditions in which osteopontin-a is cross-linked. A gain of function, conveyed through the sequence around the osteopontin-c splice site, is implied by the mutant M3 (see Table 3), which has the peri-junctional amino acids changed to alanines [44] and has lost almost all the metabolic effects of osteopontin-c. Further research will address the receptor interactions of osteopontin-c and osteopontin-a upstream of the distinct intracellular signaling pathways engaged by these forms.

While MCF-7 cells and ZR-75 cells are both ER-positive, we have previously shown that the expression of OPN-c is independent of the histologic type of breast cancer [4] and therefore have reason to believe that the observation described is common to all subtypes of breast cancer. This is also consistent with our meta-analysis that found (total) osteopontin expression to be independent of breast cancer subtypes, as classified by receptor status [45].

## Supporting Information

Supplement S1 (DOCX)

Supplement S2 (DOCX)

Supplement S3 (DOCX)

Supplement S4 (DOCX)
