# Supplementary material for: Energy Metabolism during Anchorage-Independence. Induction by Osteopontin-c
Source: PLoS One. 2014 Aug 26;9(8):e105675. doi: 10.1371/journal.pone.0105675 (PMC4144875; doi:10.1371/journal.pone.0105675)
Supplement: Supplement S1 — (DOCX) [file pone.0105675.s001.docx]

**Supplement S1**

Reactive oxygen intermediates support anchorage-independent expansion. The presence of hydrogen peroxide induced soft agar clone formation, whereas the peroxide scavengers glutathione and N-acetyl L-cysteine decreased it. None of these agents affected the proliferation of MCF-7 cells in culture dishes, indicating that their effects were exerted selectively on anchorage independent expansion and that inhibition was not a sign of toxicity. Peroxides may generate hydroxyl radicals. Therefore we also tested the effect of the hydroxyl radical scavenger mannitol on soft agar clone formation. This agent dose-dependently inhibited soft agar clone formation, while having no effect on proliferation (Figure S1A). To confirm the results obtained with chemical modifiers, we stably transfected MCF-7 cells with an expression construct for catalase targeted to the mitochondria or with a control vector. Consistently, the catalase over-expressing MCF-7 cells formed smaller clones in soft agar than the controls (Figure S1B). Together, these results pointed to a supportive role for peroxide signaling in anchorage independence.

We then assessed the genetic basis for the redox regulation of anchorage-independent growth. RNA was extracted from MCF-7 cells that had been grown in the presence or absence of hydrogen peroxide for 7 days in soft agar, and microarray hybridization was performed. According to gene ontology analysis, the expression of genes involved in metabolism, DNA regulation, and subcellular localization and transport was significantly increased by hydrogen peroxide (Table S1).

**Figure S1: The effects of redox modulators on soft agar clone formation. A)** MCF-7 cells were plated in soft agar. Every other day, starting on day 0 after the agar had solidified, medium was replenished containing the indicated redox modulators to reach the final concentrations specified. Pictures were taken on day 7 and the clone areas were measured using the software Metamorph. The data points indicate mean + std. For assaying proliferation (right panel), 2000 cells were plated per well of a 24-well plate in the presence or absence of the indicated redox modulators at concentrations at least as high as the highest dose used in soft agar to assure that any potential toxicity would not be missed (H_2_O_2_ = 25 μM, NAC = 4 mM, GSH = 4 mM, mannitol = 125 μM). Medium and redox modulator were exchanged every other day. The cell numbers were measured daily in quadruplicates using a Coulter Counter. **B)** Effect of mt-Catalase on soft agar clone formation by MCF-7 cells. MCF-7 cells were stably transfected with a catalase construct that is targeted to the mitochondria (mt-Catalase) or with a control vector. The cells were subjected to growth in soft agar, and the clone sizes were measured.

**Table S1: Microarray analysis of cells grown in soft agar in the presence or absence of hydrogen peroxide.** **A)** Gene ontology analysis of the peroxide effect with DAVID (the Database for Annotation, Visualization and Integrated Discovery). fdr = false discovery rate **B)** Significantly induced genes for metabolism. **C)** Significantly induced genes for DNA regulation. **D)** Significantly induced genes for subcellular localization and transport. **E)** Significantly induced genes without specific functional assignment.

Table S1A

gene ontology analysis

Table S1B

top changes metabolism

Table S1C

top changes DNA regulation

Table S1D

top changes subcellular transport

Table S1E

top changes unclassified
