# Supplementary material for: Energy Metabolism during Anchorage-Independence. Induction by Osteopontin-c
Source: PLoS One. 2014 Aug 26;9(8):e105675. doi: 10.1371/journal.pone.0105675 (PMC4144875; doi:10.1371/journal.pone.0105675)
Supplement: Supplement S2 — (DOCX) [file pone.0105675.s002.docx]

**Supplement S2**

**Figure S2: Real-time RT-PCR confirmation of the microarray analysis.** For select genes, identified in the microarray analysis as having altered expression induced by osteopontin-c or hydrogen peroxide (left panel), the amounts of RNA message were determined by real-time RT-PCR (right panel). V = MCF-7 vector cells, A = MCF-7 OPNa cells, C = MCF-7 OPNc cells, AC = MCF-7 OPNa+OPNc cells, UT = untreated MCF-7 cells, H_2_O_2_ = MCF-7 cells treated with hydrogen peroxide. Whereas GNB2 and PGD are expressed at very low levels in adherent cells and display substantial induction in soft agar, SWI/SNF and TH1L also have substantial expression levels in cells plated on plastic. A repeat experiment gave similar results.

Figure S2
