# Supplementary material for: Energy Metabolism during Anchorage-Independence. Induction by Osteopontin-c
Source: PLoS One. 2014 Aug 26;9(8):e105675. doi: 10.1371/journal.pone.0105675 (PMC4144875; doi:10.1371/journal.pone.0105675)
Supplement: Supplement S3 — (DOCX) [file pone.0105675.s003.docx]

**Supplement S3**

**Figure S3: RNASeq of deadherent MCF-7 transfectants.** MCF-7 cells transfected with vector, osteopontin-a, or osteopontin-c were cultured and plated in poly-HEMA coated 6-well dishes at 4x10^5^ cells for two days. RNA was extracted and processed for RNASeq. **A)** Heat map for the comparison of MCF-7 vector versus MCF-7 OPNa versus MCF-7 OPNc. The individual columns represent 3 replicates for each transfectant **B)** Volcano plots for pairwise comparisons among the transfectants. A_V = osteopontin-a versus vector, C_A = osteopontin-c versus osteopontin-a, C_V = osteopontin-c versus vector.


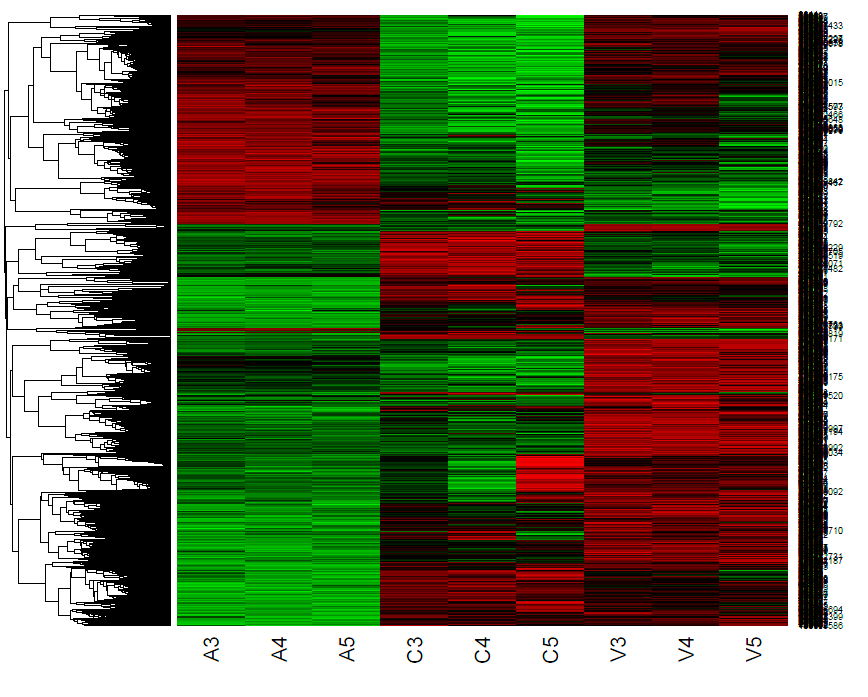

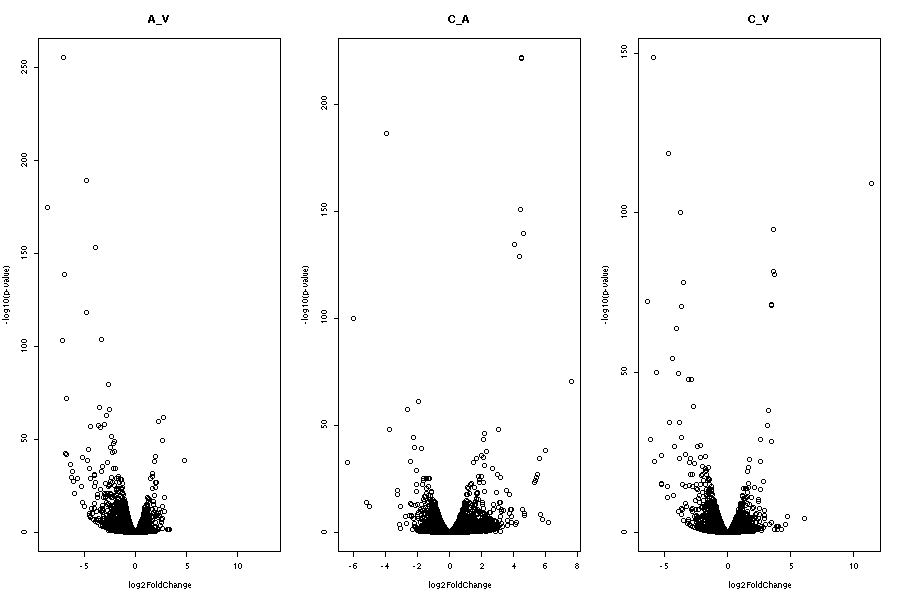


**B**

**A**
