# Supplementary material for: Energy Metabolism during Anchorage-Independence. Induction by Osteopontin-c
Source: PLoS One. 2014 Aug 26;9(8):e105675. doi: 10.1371/journal.pone.0105675 (PMC4144875; doi:10.1371/journal.pone.0105675)
Supplement: Supplement S4 — (DOCX) [file pone.0105675.s004.docx]

**Supplement S4**

According to co-expression analysis in Oncomine [41], osteopontin is upregulated together with genes of the lactate and pyruvate metabolism, as well as genes for lactate/pyruvate and glucose transporters in various cancers (Table S4).

**Table S4: Osteopontin-induced metabolic changes in cancer cells.** Gene co-expression with osteopontin according to Oncomine. Co-expression analysis was performed in Oncomine for genes that are regulated in conjunction with osteopontin (SPP1). The intensity of the yellow background reflects the strength of the correlation.

Table S4
